# Chronic Exposure to Fluoride Affects GSH Level and NOX4 Expression in Rat Model of This Element of Neurotoxicity

**DOI:** 10.3390/biom10030422

**Published:** 2020-03-09

**Authors:** Karolina Dec, Agnieszka Łukomska, Karolina Skonieczna-Żydecka, Karolina Jakubczyk, Maciej Tarnowski, Anna Lubkowska, Irena Baranowska-Bosiacka, Daniel Styburski, Marta Skórka-Majewicz, Dominika Maciejewska, Izabela Gutowska

**Affiliations:** 1 Department of Human Nutrition and Metabolomics, Pomeranian Medical University in Szczecin, Broniewskiego 24 Str., 70-460 Szczecin, Poland; karolinatdec@gmail.com (K.D.); agnieszka_lukomska@wp.pl (A.Ł.); jakubczyk.kar@gmail.com (K.J.); dmaciejewska.pum@gmail.com (D.M.); 2 Laboratory of Neuroplasticity, Nencki Institute of Experimental Biology, Polish Academy of Sciences, 3 Pasteur Street, 02-093 Warsaw, Poland; 3 Department Physiology, Pomeranian Medical University in Szczecin, Powstańców Wlkp. 72 av., 70-111 Szczecin, Poland; maciej.tarnowski@pum.edu.pl; 4 Department of Functional Diagnostics and Physical Medicine, Faculty of Health Sciences, Pomeranian Medical University in Szczecin; Żołnierska 54, 71-210 Szczecin, Poland; 5 Department of Biochemistry, Pomeranian Medical University in Szczecin, Powstańców Wlkp. 72 av., 70-111 Szczecin, Poland; ika@pum.edu.pl; 6 Department of Medical Chemistry, Pomeranian Medical University in Szczecin, Powstańców Wlkp. 72 av., 70-111 Szczecin, Poland; daniel.styburski@interia.pl (D.S.); marta_skorka@o2.pl (M.S.-M.); gutowska@pum.edu.pl (I.G.)

**Keywords:** brain, fluoride, GSH, NOX4, neurotoxicity, oxidative stress

## Abstract

Exposure of neural cells to harmful and toxic factors promotes oxidative stress, resulting in disorders of metabolism, cell differentiation, and maturation. The study examined the brains of rats pre- and postnatally exposed to sodium fluoride (NaF 50 mg/L) and activity of NADPH oxidase 4 (NOX4), catalase (CAT), superoxide dismutase (SOD), glutathione peroxidase (GPx), glutathione reductase (GR), concentration of glutathione (GSH), and total antioxidant capacity (TAC) in the cerebellum, prefrontal cortex, hippocampus, and striatum were measured. Additionally, NOX4 expression was determined by qRT–PCR. Rats exposed to fluorides (F-) showed an increase in NOX4 activity in the cerebellum and hippocampus, a decrease in its activity in the prefrontal cortex and hippocampus, and upregulation of NOX4 expression in hippocampus and its downregulation in other brain structures. Analysis also showed significant changes in the activity of all antioxidant enzymes and a decrease in TAC in brain structures. NOX4 induction and decreased antioxidant activity in central nervous system (CNS) cells may be central mechanisms of fluoride neurotoxicity. NOX4 contributes to blood–brain barrier damage, microglial activation, and neuronal loss, leading to impairment of brain function. Fluoride-induced oxidative stress involves increased reactive oxygen speciaes (ROS) production, which in turn increases the expression of genes encoding pro-inflammatory cytokines.

## 1. Introduction

The accumulation of fluoride (F-) in the body is particularly harmful to the central nervous system (CNS) of both humans and animals, leading to learning disabilities, memory and cognitive function impairment, and behavioral disorders in both young and adult individuals [1]. However, weaker protective mechanisms and enhanced blood–brain barrier (BBB) permeability make young individuals particularly vulnerable to F- damaging effects [2,3,4]. For example, in areas where fluoride concentration in drinking water significantly exceeds WHO standards, children are reported to have significantly lower (intelligence quotient) IQ scores compared to children living in uncontaminated areas [5,6,7]. The exposure of this element during pregnancy, development, and thereafter can adversely affect the brain functions of offspring [8]. A number of histopathological changes, including demyelinization, a decrease in the number of Purkinje cells, thickening and loss of dendrites, swelling of mitochondria, and dilation of endoplasmic reticulum in neurons, have been observed in the brains of experimental animals subjected to this element [9]. Alterations in the density of neurons and in the number of undifferentiated neurons have also been observed in the brains of fetuses aborted therapeutically in a geographic region characterized by endemic fluorosis [10]. Moreover, a decrease in the number of neuronal nicotinic acetylcholine receptor (nAChR) binding sites and a selective decrease in the levels of the receptor subunit proteins in PC12 cells subject to fluoride toxicity were noted. Because nAChRs play major roles in cognitive function, including learning and memory, as well as exerting a neuroprotective effect, such decreases in the number of these receptors may be an important factor in connection with the dysfunction of the central nervous system caused by F- toxicity [11].

Another important mechanism of fluoride-induced CNS impairment is oxidative stress caused by increased synthesis of reactive oxygen species (ROS), weakening of antioxidant defense mechanisms, and induction of lipid and protein oxidation [1,8,12,13,14,15,16]. ROS are highly reactive oxygen derivatives, including the superoxide radical (O_2_^−^), hydroxyl radical (OH), hydroxyl anion (OH^−^), diatomic oxygen (O_2_), and hydrogen peroxide (H_2_O_2_) [17]. Free radicals in cells are mainly generated by redox reactions catalyzed by NADPH oxidase (NOX), xanthine oxidase (XO), flavin oxidase, cytochrome P450, and by respiratory chain components in mitochondria [18]. Intracellular defense mechanisms against elevated concentrations of ROS mainly involve the action of antioxidant enzymes, including superoxide dismutase (SOD), catalase (CAT), glutathione peroxidase (GPx), and glutathione reductase (GR) [17,18].

The CNS is characterized by a high prevalence of oxygen-dependent processes, while simultaneously containing high levels of readily oxidized fatty acids and relatively low activity of antioxidant enzymes [8,19]. This creates conditions wherein exposure of neural cells to harmful factors may easily lead to the initiation of oxidative stress—an imbalance between ROS synthesis and antioxidant enzyme activity [15,20]. Under physiological conditions, ROS act as signaling molecules in the regulation of numerous processes such as gene expression, cell proliferation, cell viability, apoptosis, and immune response to external factors [17]. An increase in ROS synthesis may, therefore, influence the rate of consumption of substrates or cofactors necessary for the proper functioning of the antioxidant enzymes responsible for their removal, thereby inducing oxidative stress and leading to disturbances in cell metabolism and damage to the entire brain [13,17,21,22,23].

Given the aforementioned evidence of a role for fluoride-induced oxidative stress in CNS impairment, this study aims to further elucidate the underlying mechanisms by analyzing the effect of pre- and postnatally administered low doses of F- on the activity of enzymes responsible for free radical processes in the cerebellum, prefrontal cortex, hippocampus, and striatum of rats.

## 2. Materials and Methods

### 2.1. Animal Procedures

This study was performed on brain tissues from rats exposed pre- and postnatally to sodium fluoride (NaF). Animal procedures were carried out in strict accordance with international standards of animal care, and every effort was made to minimize suffering and the number of used animals. Experiments were approved by the Local Ethical Committee on Animal Testing in Szczecin, Poland (approval No. 32/2015). All applicable international, national, and/or institutional guide-lines for the care and use of animals. All animals were given access to food (standard diet) and drinking water ad libitum. The cages were kept in a controlled temperature environment on a 12-h/12-h light/dark schedule.

Pregnant females Wistar rats were randomly divided into two groups—control and fluoride. Animals from the control group (*n* = 6) received tap water to drink, while animals from the experimental group (fluoride, *n* = 6) received drinking water containing NaF in concentration 50 mg/L from pregnancy day 0 to postnatal day 90 (PND 90). Pups were separated from their mothers at PND 21 (end of breast-feeding) and were kept under the same conditions as previously described until reaching maturity (PND 90) [24]. All animals were sacrificed by decapitation; brain structures were dissected and placed in liquid nitrogen. Samples were stored at −80 °C for later analysis.

Oral administration of NaF has been chosen in this experimental design as it reflects human environmental exposure. Rats consume between 30 to 50 mL of water daily, which when given 50 mg/L, gives an intake of 1.5 mg up to 2.5 mg of F^−^ throughout the day. The consumption norms of F- according to the Polish standards SAI (safe and adequate daily intake) as well as ADI (acceptable daily intake) amount to 3–4 mg/day for an adult (depending on gender). Environmental studies have shown that the symptoms of fluorosis in an adult human weighing 70 kg appear with a consumption of more than 10 mg of F^−^ per day [24].

### 2.2. Measurement of NOX4 Concentration

Analysis of NOX4 concentration was performed by ELISA using Rat NADPH Oxidase 4 ELISA Kit (EIAab, Wuhan, China). The material was prepared and tested according to the manufacturer’s recommendations. Measurement was performed on the ASYS UVM 340 spectrophotometer (Biogenet, Parkingowa, Poland).

### 2.3. Analysis of NOX4 Gene Expression by qRT-PCR

Analysis of *NOX4* expression was performed by quantitative Reverse Transcription Polimerase Chain Reaction (qRT–PCR). Following dissection, brain tissues were immediately placed in the RNAlater buffer (Qiagen, Wrocław, Poland) to inhibit RNA degradation. RNA was extracted from tissue samples using an RNeasy Lipid Tissue Mini Kit (Qiagen, Poland), according to the manufacturer’s instructions. Then, 1 μg of extracted RNA was prepared for analysis using a FirstStrand cDNA synthesis kit and oligo-dT primers (ThermoFisher, Warszawa, Poland). To quantify mRNA levels, qRT–PCR was performed using an ABI 7500Fast and Power Master SYBR Green PCR Master Mix (ThermoFisher, Poland). The following primer pairs were used: *GAPDH* forward: ATGACTCTACCCACGGCAAG, reverse: CTGGAAGATGGTGATGGGTT; *NOX4* forward: AGTCAAACAGATGGGA, reverse: TGTCCCATATGAGTTGTT.

### 2.4. Measurement of Antioxidative Enzyme Activity and GSH Concentration

The activities of SOD, CAT, GPx, GR, as well as total GSH levels in rat brain structures, were measured using assay kits from Cayman Chemical Company (Biokom, Janki, Poland): Superoxide Dismutase Assay Kit, Catalase Assay Kit, Glutathione Peroxidase Assay Kit, Glutathione Reductase Assay Kit, and Glutathione Assay Kit. All procedures were carried out in accordance with the manufacturer’s recommendations. Measurement was performed with the ASYS UVM 340 (Biogenet, Poland) spectrophotometer [25,26,27,28,29].

### 2.5. Measurement of TAC

TAC was measured using the Antioxidant Assay Kit (Cayman Chemical Company, Biokom, Poland) and the ASYS UVM 340 spectrophotometer (Biogenet, Poland). This kit measures the content of both water-soluble and lipid-soluble antioxidants; the result obtained includes antioxidant enzymes as well as vitamins, lipids, glutathione, uric acid, and other antioxidant molecules in the test sample. The procedures were performed in accordance with the manufacturer’s recommendations [30].

### 2.6. Measurement of Protein Concentration

Protein concentration was determined spectrophotometrically using the MicroBCA Protein Assay Kit (ThermoFisher, Poland). Sample preparation was performed in accordance with the manufacturer’s recommendations. Measurement was performed with an ASYS UVM 340 spectrophotometer (Biogenet, Poland) and the results were read in MicroWIN (Microwin Technology Solutions Limited, Hong Kong). Protein concentration was calculated based on the obtained standard curve.

### 2.7. Statistical Analysis

Obtained results were analyzed statistically using a Statistica 12.0 package (StatSoft, Dell, Round Rock, TX, USA). For each of the examined parameters, the arithmetic mean ± standard deviation (SD) was calculated. The Shapiro–Wilk (W) test was used to obtain the distribution of results for individual variables. Most of the data differed from the normal distribution; therefore, non-parametric tests were used for further analysis. The Mann–Whitney *U*-test was used to assess the differences between the control group and the study group. Differences were deemed statistically significant at *p* ≤ 0.05.

## 3. Results

### 3.1. Effects of F- Exposure during Pre- and Postnatal Development on NOX4 Protein Concentration and Gene Expression in Rat Brain Structures

NOX are endothelial proteins responsible for significant ROS production in cells. In order to determine the contribution of isoform 4 NOX to free radical processes in the brain, protein concentration and gene expression in the cerebellum, prefrontal cortex, hippocampus, and striatum of rats from the control group, subjected to pre- and postnatal F- exposure, were measured.

**Figure 1:**
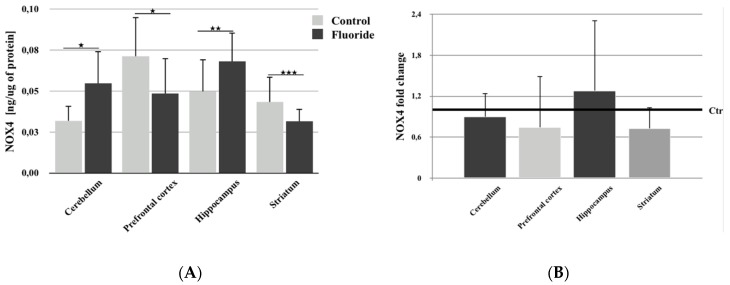
The concentration of NADPH oxidase 4 (NOX4) (**A**) and its expression (fold change) (**B**) in studied brain structures (prefrontal cortex, cerebellum, hippocampus, and striatum) in control (Ctr; *n* = 6) and F-exposed group (F) (*n* = 6). * *p* ≤ 0.05, ** *p* ≤ 0.01, *** *p* ≤ 0.001 for the significance of difference between the groups (Mann–Whitney test).

There was a statistically significant increase in NOX4 protein concentration in the cerebellum (+71.9%; *p* = 0.011) and hippocampus (+36.0%; *p* = 0.009) in comparison to control, while a statistically significant decrease in enzyme protein concentration occurred in the prefrontal cortex (−30.9%; *p* = 0.015) and striatum (−25.6%; *p* = 0.00005) (Figure 1A).

qRT–PCR analysis of *NOX4* expression showed no statistically significant changes in NOX4 expression in studied brain structures (Figure 1B). Non-significant upregulation of the gene expression was observed in the hippocampus, and its downregulation was observed in the prefrontal cortex, cerebellum, and striatum (Figure 1B).

### 3.2. Effects of Fluoride on SOD, CAT, GPx, and GR Activity and GSH Concentration in the Rat Brain

**Figure 2:**
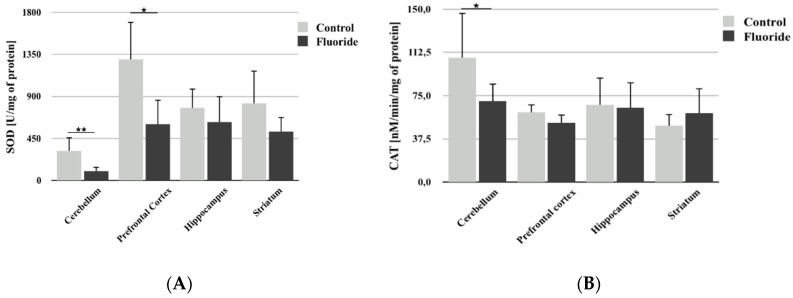
The effect of pre- and postnatal exposition to NaF on superoxide dismutase (SOD) (**A**) and catalase (CAT) (**B**) activity in different rat brain structures (prefrontal cortex, cerebellum, hippocampus, and striatum) in control (*n* = 6) and F-exposed group (F) (*n* = 6). * *p* ≤ 0.05, for the significance of difference between the groups (Mann–Whitney test).

Analysis of SOD activity showed that pre- and postnatal fluoride exposure leads to a decrease in its activity only in the cerebellum (−68.2%; *p* = 0.005) and prefrontal cortex (Figure 2A). In the hippocampus and striatum, measured enzyme activity was lower, but the differences were not statistically significant.

A significant decrease in CAT activity was observed following fluoride exposure in the cerebellum (−54.2%; *p* = 0.043) (Figure 2B). No statistically significant differences were observed in other brain structures studied.

**Figure 3:**
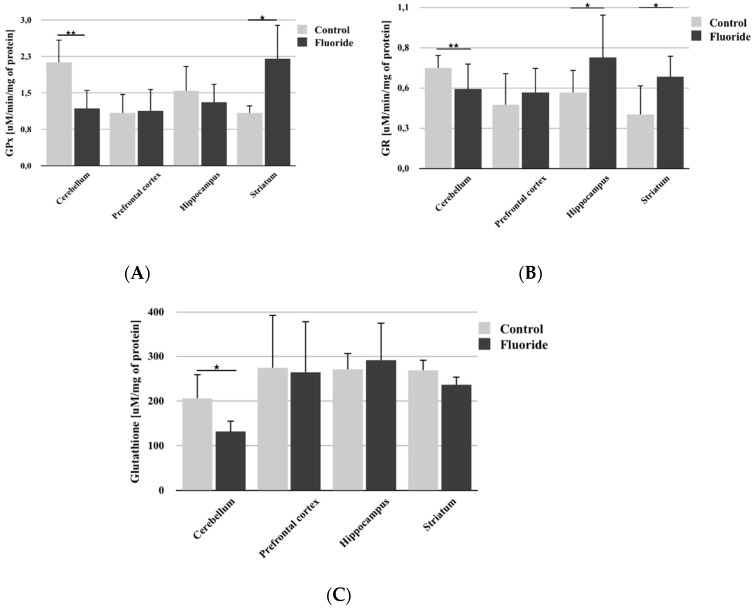
The effect of pre- and postnatal exposition to NaF on glutathione peroxidase (GPx) activity (**A**), glutathione reductase (GR) activity (**B**), and glutathione (GSH) concentration (**C**) in different rat brain structures (prefrontal cortex, cerebellum, hippocampus, and striatum) in control (*n* = 6) and F-exposed group (F) (*n* = 6). * *p* ≤ 0.05, ** *p* ≤ 0.01, for the significance of difference between the groups (Mann–Whitney test).

Analysis of GPx activity showed a statistically significant decrease in the cerebellum (−44.8%; *p* = 0.003; Figure 3A) and a significant increase in the striatum (+102.7%; *p* = 0.036) compared to control. No significant differences in activity were found in the other brain structures studied.

Fluoride exposure led to a slight but statistically significant decrease in GR activity (−20.6%; *p* = 0.008) in the rat cerebellum (Figure 3B). In the other examined brain structures, an increase in enzyme activity was observed, but this increase was statistically significant only in the hippocampus (+46.1%; *p* = 0.031) and striatum (+72.2%; *p* = 0.034).

Analysis also showed a small but statistically significant decrease in GSH concentration in the cerebellum (−36.2%; *p* = 0.029); no statistically significant differences were found in other brain structures (Figure 3C).

### 3.3. Reduction in TAC as a Result of Chronic Exposure to Fluoride during pre- and Postnatal Development

**Figure 4:**
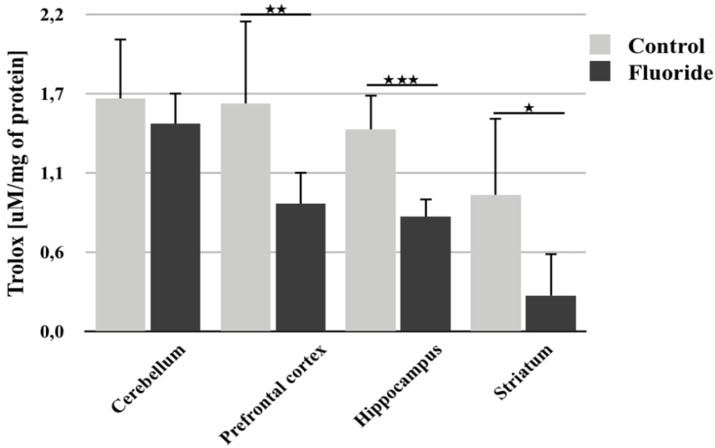
The total antioxidant capacity (TAC) of the tissue, measured in different rat brain structures (prefrontal cortex, cerebellum, hippocampus, and striatum) in control (*n* = 6) and F-exposed group (F) (*n* = 6). * *p* ≤ 0.05, ** *p* ≤ 0.01, *** *p* ≤ 0.001 for the significance of difference between the groups (Mann–Whitney test).

Analysis of total antioxidant capacity following perinatal exposure to F- showed a statistically significant decrease in TAC in the prefrontal cortex (−43.7%; *p* = 0.002), hippocampus (−43.6%; *p* = 0.0001) and striatum (−73.7%; *p* = 0.043) (Figure 4). Cerebellar TAC also decreased, but the change was not statistically significant.

## 4. Discussion

The synthesis of ROS, which, under physiological conditions, act as signaling molecules, is regulated in vivo by pro- and antioxidant enzymes and molecules. Disturbances in this balance lead to oxidative stress. The brain is particularly sensitive to oxidative stress due to its high O_2_ consumption and relatively low levels of antioxidant enzyme activity. Additionally, the brain contains a large number of polyunsaturated fatty acids (including membrane phospholipids, which can easily undergo oxidation induced by free radicals) and high concentrations of Fe^2+^, Cu^2+^, and Zn^2+^ ions, especially in substantia nigra and striatum [17,31,32]. Changes in levels of neuronal membrane phospholipids, due to their oxidation and release from the membrane, result in changes in cell membrane fluidity, stability, and permeability [9,33]. Moreover, oxidized membrane lipids can be transformed into biologically active compounds involved in the development of inflammation (e.g., prostanoids, leukotrienes, and lipoxins synthesized in cyclooxygenase and lipoxygenase pathways) [34]. On the other hand, ROS themselves also can induce inflammatory processes by activating NF-κB-dependent transcription of inflammatory factors [35].

Previous in vitro and in vivo studies have confirmed that fluoride accumulation in the brain leads to an increase in ROS concentration, decrease antioxidant enzyme activity, and an increase in lipid peroxidation. Throughout literature, data clearly indicate that exposure to both low and high F- concentrations promotes the synthesis of ROS and malondialdehyde (MDA, a marker of oxidative stress) in the brain [15,16,35,36]. Most studies also confirm that long-term exposure leads to a decrease in the activity of the antioxidant enzymes responsible for maintaining proper redox status in cells, although this depends on fluoride concentration, exposure period, examined brain structures, and animal age [16,37,38].

F- may indirectly induce inflammation in the brain by promoting oxidative stress, which in turn increases the synthesis of pro-inflammatory molecules. Chronic inflammation in CNS has been implicated in the loss of neurons in neurodegenerative diseases [39].

### 4.1. The Effect of Perinatal Exposure to Fluoride on the Expression and Activity of NOX4 in the Rat Brain

ROS are produced in large quantities in the mitochondria during electron transport chain reactions through mitochondrial membrane complexes. Complexes I, II, and III may “leak” electrons, reducing oxygen molecules to reactive superoxide O_2_^−^, which are then converted to H_2_O_2_ [40]. Another important source of ROS is enzymatic NOX-catalyzed reactions occurring in various cellular compartments [41,42]. The function of NOX varies depending on its location in cells and its level of activity. In the CNS under physiological conditions, ROS produced by these enzymes are mainly responsible for the regulation of inflammatory processes (including microglia activation), cellular signaling, posttranslational modification of proteins, regulation of gene expression, and processes such as apoptosis and neuroplasticity [43,44].

NOX4, an enzyme responsible for the synthesis of H_2_O_2_ from molecular oxygen, is, like all NOX isoforms, a membrane-bound enzyme. It occurs in the endoplasmic reticulum, mitochondria, and perinuclear space, but not in the outer cell membrane [45,46,47,48]. In the CNS, NOX4 is expressed in the cerebral cortex, hippocampus, and cerebellum [49]. So far, it has been confirmed that NOX4 is responsible for a significant generation of ROS in the brain and an increase in its activity has been implicated in the development of neurodegenerative diseases, both acute and chronic [49,50].

In this study, we examined the expression level of *NOX4* and observed that it was additionally expressed in the striatum, where its level was comparable to other tested brain structures. Analysis of NOX4 protein concentration showed that perinatal exposure to F- lead to increased protein expression in the hippocampus and cerebellum, which may be connected with the induction of H_2_O_2_ synthesis in the cerebellum and hippocampus. A significant decrease in NOX4 concentration was observed in the prefrontal cortex and striatum of exposed rats. Pre- and postnatal exposure to fluoride (50 mg/L) lead to the downregulation of *NOX4* expression in all brain structures besides the hippocampus, where upregulation was observed. The observed changes in *NOX4* expression and protein concentration in the cerebellum indicate a probable feedback mechanism inhibiting *NOX4* expression. It is likely that fluoride passing through the blood–brain barrier (BBB) promotes enzymatic activity in the examined brain structures, while the increasing concentration of H_2_O_2_ reduces gene expression. This is supported by the observed increase in NOX4 protein concentration and decrease in gene expression in the cerebellum of fluoride-exposed individuals. In the prefrontal cortex and striatum of exposed rats, the observed decrease in protein concentration may indicate a faster mechanism of response to the increasing concentration of H_2_O_2_, resulting in the inhibition of gene expression.

Since H_2_O_2_ produced by NOX4 is uncharged, it can freely penetrate biological membranes and act as a signaling molecule. Conversely, it can directly damage cells by oxidation of nucleic acids and lipids, especially at excessive concentrations [49,51]. As mentioned earlier, the function of NOX4 depends on, among other things, its location in the cell. The isoform present in the nucleus and nucleolus is linked to regulating the expression of genes associated with the response to oxidative stress as well as participating in oxidative DNA damage and apoptosis initiation through caspase-3 activation, resulting in loss of neurons [52,53,54]. Therefore, an increase in NOX4 activity in rat brain structures exposed to F- likely influences the regulation of free-radical processes and the induction of proinflammatory cytokine synthesis by initiating changes in gene expression [54]. Casas et al. presented a cell-specific system in which induction of NOX4 in the endothelial cells forming the BBB leads to damage and increased flow of proinflammatory and toxic factors into the brain, while the concurrent increase in enzyme activity in neuronal cells leads to autotoxicity [55]. Our analysis showed a significant increase in NOX4 protein expression in the hippocampus and cerebellum- structures responsible for the consolidation of short-term to long-term memory, numerous cognitive functions, spatial orientation, and motor control. As young individuals are more susceptible to NOX4-mediated damage due to weaker protective mechanisms and enhanced blood–brain barrier (BBB) permeability, exposure to F- seems to be particularly dangerous, as it can lead to permanent damage and disrupted development of the aforementioned essential brain structures.

### 4.2. The Effect of Perinatal Fluoride Exposure on the Activity of Antioxidant Enzymes and GSH Concentration in the Rat Brain

Though ROS can act as signaling molecules and regulate cellular processes, excessive concentrations cause damage to cellular structures, including DNA, lipids, and proteins, and disrupt tissue function. Living organisms have developed protective mechanisms against the harmful effects of ROS utilizing intracellular enzymatic systems, which involve low-molecular weight molecules such as GSH, vitamin C, and coenzyme Q, and macromolecular antioxidant enzymes (i.e., CAT, SOD, GPx, and GR) [56]. These antioxidant enzymes work collaboratively and are essential for the proper functioning of the cell.

The present analysis focuses on the influence of F- exposure during development on the activity of CAT, SOD, GPx, and GR in the rat brain. Our results found a statistically significant decrease in the activity of all the examined enzymes—CAT, SOD, GPx, and GR—in the cerebellum. Conversely, in the prefrontal cortex, a decrease in activity was observed only with SOD. Other examined structures (hippocampus and striatum) only showed a significant increase in GR activity and a slight decrease in SOD activity. Additionally, a decrease in TAC was observed in all brain structures studied, which was statistically significant in all structures except the cerebellum. The inhibition of antioxidant enzymes (SOD, CAT) activity in the brains of mice treated with F- was previously demonstrated by Vani and Reddy [57].

The observed changes in the activity of antioxidant enzymes may indicate an intensification of ROS synthesis in the examined brain structures. Increased activity of GR in the hippocampus and striatum, indicates increased activation of mechanisms for the removal of harmful radicals. However, the decrease in SOD activity in all examined structures and in CAT, GPx, and GR activity in the cerebellum, may indicate the consumption of substrates or cofactors necessary for these enzymes’ action due to the increasing synthesis of ROS and subsequent alterations in cell signaling. Changes in enzyme activity may also be related to the formation of insoluble complexes of F- with cations in the active sites of these enzymes, leading to inhibition of their activity [58]. Additionally, fluoride may associate with functional groups of amino acids surrounding the enzyme active site, changing its conformation and inhibiting its action [59,60]. Our findings in the cerebellum indicate that the decrease in GPx and GR activity is most likely due to the decrease in the concentration of GSH, an essential cofactor for the action of these enzymes. It is likely that F- disturbs the activity of enzymes involved in the synthesis of GSH, leading to a decrease in its concentration and in the activity of GPx and GR. This study also found that the activity of individual enzymes differed between brain areas and that these changes were not unidirectional within a given area. However, TAC analysis confirmed a universal decrease in antioxidant activity following long-term exposure to F-.

Numerous studies have shown that oxidative stress contributes to the development of neurodegenerative and demyelination diseases, including Alzheimer’s disease (AD), Parkinson’s disease (PD), Huntington’s disease (HD), and multiple sclerosis (MS) [19]. In agreement with our results, both in animal models and patients, observed changes in individual antioxidant enzyme activities are not unidirectional. In patients with AD, researchers report an increase in SOD activity in the prefrontal cortex and an increase in enzyme activity in the hippocampus and caudate nucleus. However, Murakami et al., using a mouse model of AD, showed that the induction of inflammation, phosphorylation of Tau protein, and production of abnormal amyloid was associated with a decrease in SOD activity. It is widely assumed that increased activity of antioxidant enzymes in individual regions of the brain in AD patients represents a mechanism to compensate for increased oxidative stress [61,62]. Like in AD patients, our analysis of perinatal neurotoxicity of F- in rats showed multidirectional changes in the activity antioxidants. Therefore we deduce that, like in AD, increases in the activities of individual enzymes may be caused by an attempt to compensate for enhanced oxidative stress in a given brain structure, while decreases in their activities may be due to a direct inhibitory effect of fluoride on enzyme activity, the unavailability of cofactors necessary for their action, or downregulation through feedback [58,62,63]. Such changes in the activity of antioxidant systems resulting from exposure to F- may disturb CNS homeostasis and contribute to impaired development of young individuals [64].

## 5. Conclusions

The obtained results clearly show that exposure to F- led to an imbalance between ROS synthesis and the activity of antioxidant enzymes in the brain. This is indicated by an increase in the concentration of NOX4 (participating in the synthesis of H_2_O_2_) and a decrease in TAC in the studied brain structures. Our analysis confirms previous reports that F- inhibits the activity of antioxidant enzymes in the brain not only directly—by binding to elements in the enzyme’s active site—but also indirectly by promoting the consumption of cofactors necessary for their action. Cofactor consumption results from continuous induction of ROS synthesis, as indicated by reduced GSH levels and increased NOX4 protein concentration. The role of ROS in the induction of expression of genes encoding pro-inflammatory cytokines makes oxidative stress one of the main factors in promoting pathological inflammatory states.

We have previously demonstrated that long-term exposure of rats to NaF 50 mg/L in drinking water affects lipid metabolism in the liver and brain [24,65]. Using the same experimental model as presented in this study, we found that pre- and postnatal exposure to F- causes irreversible changes in the liver. Morphological changes resembling early phases of steatosis, which indicate the first phase of non-alcoholic fatty liver disease, were observed in rat liver [24]. Using the experimental model described in this study, we have also found that F- lead to changes in lipid metabolism in the brain and the structure especially vulnerable to its action is the hippocampus. Long term exposure to this element cause changes in the activity of enzymes implicated in lipid metabolism, which affect arachidonic acid metabolites—prostaglandin E2 and thromboxane B2, concentration in the brain [65]. The mammalian brain, in comparison to other tissues, is highly enriched in lipids that are vulnerable to oxidative stress. Under pathological conditions when ROS synthesis in the brain is increased, lipid oxidation may trigger local inflammation.

Overall, the oxidative imbalance observed in the studied brain structures, together with previously published findings, show that exposure to fluoride in the period from prenatal development to full sexual maturity can lead to irreversible and detrimental changes in rat brain.

## Abbreviations

BBB	blood-brain barrier
CAT	catalase
CNS	central nervous system
GPx	glutathione peroxidase
GR	glutathione reductase
GSH	glutathione
MDA	malondialdehyde
NF-kB	nuclear factor kappa-light-chain-enhancer of activated B cells
NOX	NADPH oxidase
PND	postnatal day
qRT-PCR	quantitative Reverse Transcription Polimerase Chain Reaction
ROS	reactive oxygen species
SOD	superoxide dismutase
TAC	total antioxidant capacity
